# The Treatment of Putrescent Pulps

**Published:** 1893-03

**Authors:** Arthur L. Swift

**Affiliations:** New York


### THE TREATMENT OF PUTRESCENT PULPS.[1]

BY DR. ARTHUR L. SWIFT, NEW YORK.

THE subject assigned for this paper, "The Treatment of Putrescent Pulps," is one which has been largely written upon, and the many methods of treatment and numerous drugs employed are as familiar to us all as is the fragrance of sulphuretted hydrogen, the invariable accompaniment; hence it is not the intention of the writer to burden you with a general discussion of such well-known methods, or with the consideration of their respective merits, but

---

[1] Read before the Central Dental Association of Northern New Jersey, December 19, 1892.

simply to describe what has proved the most successful method in his practice.

I have long since discarded the use in pulp-canals of coagulants, such as carbolic acid, creosote, etc., upon the theory that the coagulum dams up the tubuli and thus prevents diffused medication and thorough disinfection, and instead employ the diffusible essential oils, such as eucalyptus, eugenol, oil of cassia, myrtol, etc., which are very diffusible, carrying large quantities of oxygen and depositing volatile camphors, which are all-powerful in the destruction of septic and infectious matter.

Oxygen stored up in the tubuli aids by its bleaching qualities in retaining the natural color of the tooth, and the non-irritating effects of these oils, with their gentle stimulating action, marked diffusibility, and powerful disinfectant properties, make them especially valuable in these putrescent and inflamed conditions.

In this the age of antiseptic surgery, it behooves us to use every possible aseptic precaution; consequently, care should always be taken from first to last to exclude all saliva, and no instruments should be used which have not been thoroughly sterilized. Having gained free access to the pulp-chamber, wipe out the cavity with cotton saturated with equal parts of peroxide of hydrogen and bichloride of mercury, 1 to 1000, and carefully remove the contents of the canal so as to avoid penetrating the apex, or forcing any septic matter through it; then wipe out the canal with cotton on a broach, saturated with peroxide of hydrogen and bichloride solution, and dry thoroughly with bibulous paper or cotton, followed by use of hot air, and pump into the canal oil of cassia, eugenol, or myrtol, and insert very loosely a whisp of cotton or floss silk saturated with oil of cassia, and seal with gutta-percha, perforating the filling, and if periosteal inflammation exists, paint the gums with saturated tincture of aconite and iodine, and dismiss the case for three or four days. When next seen remove the dressing and pump eucalyptol or myrtol freely into the canal with cotton on a broach, and follow with a dressing of cotton or silk saturated with eucalyptol packed tightly and sealed with gutta percha without perforation, and dismiss for about ten days. When next seen the canal will be in good condition for filling. Then treat as when last seen, pumping eucalyptol or myrtol, or equal parts of both, into the canal; and after thoroughly drying fill the canal with oxychloride or chloro-percha.

In case of blind abscess, drain the abscess thoroughly by frequent wipings of peroxide of hydrogen and bichloride solution, and inject without pressure oil of cassia, or equal parts of cassia and eucalyptol, and insert a whisp of cotton saturated with the above very loosely, not closing apex, and seal with gutta-percha and perforate, painting gums with tincture of aconite and iodine. Examine it again in about four days and treat as before, packing dressing, saturated with eucalyptol or myrtol, and seal tightly; and if after ten days tooth is not sore to percussion, fill the canal. If, after having opened into the canal, pain and inflammation continue for some time without abatement, I have found it successful to open into the apical space with a small trephine, which causes very little pain with the use of local anæsthetics, and treat with peroxide and bichloride solution, injecting eucalyptol or myrtol, placing a tent of cotton moistened with oil of cassia in the opening, and treating the canal as before.

In case of abscess with fistula, syringe through it, and inject oil of cassia or eugenol through the canal until it comes out at the opening; dress as before with cassia or eugenol, putting a tent of cotton in the fistula saturated with cassia, and seal with Fowler's stopping or gutta-percha and perforate. Examine it in two days; treat the canal as before, and pack tightly a dressing of eucalyptol or myrtol; seal without perforation. Examine further in two days, and then pump eucalyptol into the canal; dry and fill the canal, and treat the fistula with cassia, and examine it occasionally until healed.

If after filling roots in any of the conditions described trouble should subsequently occur, which has not been the case in the writer's practice during a period of about six years, a trephine should be used, opening into apical space, and treating through the opening, upon the theory that the tooth having been thoroughly disinfected and filled, the trouble must be simply an apical pericementitis, caused by some septic matter having found its way beyond the apex, and by gaining direct access to the apical space the cause of trouble may be removed.

